# Association between Rumination Times Detected by an Ear Tag-Based Accelerometer System and Rumen Physiology in Dairy Cows

**DOI:** 10.3390/ani13040759

**Published:** 2023-02-20

**Authors:** Anne Simoni, Andrew Hancock, Christian Wunderlich, Marcus Klawitter, Thomas Breuer, Felix König, Karina Weimar, Marc Drillich, Michael Iwersen

**Affiliations:** 1 University Clinic for Ruminants, Clinical Unit for Herd Health Management in Ruminants, University of Veterinary Medicine, 1210 Vienna, Austria; 2 Zoetis International, D18 T3Y1 Dublin, Ireland; 3 Zoetis Germany GmbH, 10785 Berlin, Germany; 4 FFoQSI GmbH—Austrian Competence Centre for Feed and Food Quality, Safety and Innovation, Technopark 1D, 3430 Tulln, Austria

**Keywords:** rumen fluid, rumination, health alert, accelerometer

## Abstract

**Simple Summary:**

Rumination is important for the digestive physiology of cattle and can currently be continuously recorded by sensor technologies. Although a decrease in rumination time is associated with several disorders, it often does not lead farmers to take specific action. In this study, we monitored rumination activity with the use of an ear-tag-based accelerometer system. To investigate the association between a decrease in rumination time and the digestive physiology of dairy cows, we compared rumen fluid characteristics between cows with accelerometer-based health alerts and matched healthy counterparts. Cows with health alerts showed greater variations in rumen fluid characteristics during the health alerts than healthy cows.

**Abstract:**

Monitoring rumination activity is considered a useful indicator for the early detection of diseases and metabolic disorders. Accelerometer-based sensor systems provide health alerts based on individual thresholds of rumination times in dairy cows. Detailed knowledge of the relationship between sensor-based rumination times and rumen physiology would help detect conspicuous animals and evaluate the treatment’s success. This study aimed to investigate the association between sensor-based health alerts and rumen fluid characteristics in Holstein-Friesian cows at different stages of lactation. Rumen fluid was collected via a stomach tube from 63 pairs of cows with and without health alerts (ALRT vs NALRT). Pairs were matched based on the day of lactation, the number of lactations, and health criteria. Rumen fluid was collected during and after health alerts. The parameters of color, odor, consistency, pH, redox potential, sedimentation flotation time, and the number of protozoa were examined. Results showed differences between both groups in odor, rumen pH, sedimentation flotation time, and protozoan count at the first rumen fluid collection. Within the groups, greater variations in rumen fluid parameters were found for ALRT cows compared to NALRT cows. The interaction between health alert and stage of lactation did not affect the rumen fluid parameters.

## 1. Introduction

An important part of the rumen physiology in dairy cows is rumination, characterized by regurgitation, remastication, and reswallowing [1,2]. Rumination reduces the particle size of feed and enables the attachment and colonization for the microbiological digestion of feed particles [2,3].

Changes in rumen physiology and related disorders can be detected by the collection of rumen fluid, which can be examined for several characteristics including sensory parameters, for example, odor, color, rumen pH, redox potential, and microbiological activity [4,5]. Steiner et al. [5] developed a scoring system to assess rumen function, including the mentioned rumen fluid parameters. Steen [6] found a reduced activity and concentration of protozoa in cows with indigestion. Huang et al. [7] reported that the redox potential, measured by an electrode, is a potential indicator of rumen function.

In addition to rumen fluid samples, the use of rumination time is also considered an indicator of a healthy rumen function [8]. Research on the association between decreasing rumination time and health disorders is mainly focused on dairy cows in the transition period [9,10], which is characterized by metabolic challenges and a greater prevalence of diseases [11,12,13] than other stages of lactation. At calving, not only does the rumination behavior of cows differ [14], but also the rumen metabolism and microbial populations change from prepartum conditions [15]. This is a consequence of dietary changes to meet the increasing requirement for milk production, which challenges the rumen environment [16,17]. Thus, it can be assumed that the prognostic value of rumen fluid may change for cows from early lactation to high lactation.

Presently, rumination times can be recorded automatically and continuously by wearable precision monitoring technologies. Wearable sensors detect rumination times either through measured accelerations of a cow’s head and ear movements or the recorded sound of rumination through a microphone [18]. Several of these sensor systems with validated performance are commercially available [18]. Some of these tag-based accelerometer systems provide the feature of health alerts based on an individual change in the cow’s rumination time. Health alerts can support decision-making by farmers and can help to recognize conspicuous animals at an early stage of disease [19]. This, however, requires high accuracy of the systems [19].

The relationship between the time a cow spends ruminating detected by the use of sensor technology and several diseases has already been investigated [20,21,22]. Silva et al. [23] evaluated the diagnosis, detection, and treatment of diseases in the fresh cow period with a focus on health alerts generated by sensor technology. Another alert system based on rumination time and activity levels was evaluated by Stangaferro et al. [24]. The authors reported a sensitivity of 93% of the system to detect metabolic and digestive disorders. Rumination time measured by a microphone-based monitoring system and rumen pH has also been linked to rumen acidosis by demonstrating an association between increased rumination time after the morning feeding and greater rumen acidity [25].

In summary, research on rumination time has mainly focused on the association with different diseases, although rumination activity assumes great importance for the physiology of the rumen [3]. The authors are not aware of previous research investigating the association between sensor-based health alerts and various rumen fluid parameters in dairy cows. In consequence, it is of interest if and to what extent automated monitoring of rumination activity and health alerts reflect changes in rumen fluid parameters.

This study investigates the association between health alerts and rumen function parameters. Health alerts are generated by an artificial intelligence system, which detects deviations in individual rumination times. First, we hypothesize that the rumen fluid parameters from cows with sensor-based health alerts differ from matched cows without health alerts. Additionally, it is hypothesized that there will be differences in the rumen fluid parameters of cows with health alerts, during both rumen fluid collection times. Second, we suspect different values of rumen fluid parameters for cows with health alerts in mid to late lactation compared with cows in early lactation, caused by the metabolic and behavioral changes around calving.

## 2. Materials and Methods

### 2.1. Animals, Housing, and Feeding

All study procedures were approved by the State Office of Agriculture, Food Safety and Fisheries Mecklenburg-Vorpommern, Germany (7221.3-2-013/21), and noted by the Ethics Committee of the University of Veterinary Medicine, Vienna. The study was conducted between April and October 2021 on a conventional dairy farm in the north of Germany, housing approximately 1900 Holstein-Friesian cows.

Animals at the close-up period (21 days before expected calving) until the fresh cow period (first 10 days after calving) were housed in a barn for cows with special needs. This barn was equipped with cubicles with recycled manure solid as bedding material in the close-up group, group pens with straw bedding for calving, cubicles equipped with straw chalk bedding for the fresh cow group, and a compartment for diseased animals. Fresh cows were milked twice daily in a 12-side-by-side milking parlor. After this period, cows were integrated into groups of approximately 200 animals according to reproduction status, lactation number, and somatic cell count. The free stall barns were equipped with cubicles with straw chalk bedding or recycled manure solid. In these groups, cows were milked in a 48-side-by-side milking parlor three times per day. Diseased cows in mid to late lactation were transferred to the special needs barn and fed with the same diet as cows in early lactation.

The average energy-corrected milk yield (based on 4% butterfat and 3.4% protein) was 10,301 kg per cow in 2021. The ration consisted of a total mixed ration (TMR) based on corn silage, grass silage, concentrates (rape seed as extraction meal and expeller, soy extract grist), and dietary supplements (Table 1), offered once a day and pushed-up every four hours. During the study period, the diet composition was adjusted based on weekly analyses of the dry matter content of the main components. Cows had ad libitum access to water.

### 2.2. Health Alerts Generated by the Accelerometer-Based Monitoring System

All cows were equipped with a SMARTBOW ear tag (SMARTBOW ear tag, Smartbow/Zoetis LLC, Weibern, Austria; size and weight 52 × 36 × 17 mm and 34 g). The ear tags recorded three-dimensional acceleration data of head and ear movements with a sampling frequency of 1 Hz. The data were sent in real-time to receivers, which were connected to a local server on the farm to process and analyze the incoming data by a machine learning procedure. Algorithms of the company provided a “health alert” caused by an urgent rumination decline within the past 24 h as well as a decrease in rumination for several days. For this, artificial intelligence analyzed the acceleration data of the individual cow every 20 min and compared it with the previous hours of rumination. Additionally, the acceleration data were analyzed every hour and compared with the rumination activity of the previous 24 h and the course of rumination in the past few days. The exact operating principle of these algorithms is the intellectual property of the company. Health alerts were displayed on a computer and were sent to a mobile device.

### 2.3. Selection of Animals

Rumen fluid was collected and examined from multiparous cows at two different stages of lactation: early lactation (eL, up to 10 days in milk (DIM) *n* = 20 cows), when cows were housed in the fresh cow barn, and mid to late lactation, when cows were integrated into groups of 200 animals (mlL, from 20 DIM up to dry off; *n* = 43 cows). Cows with a health alert (ALRT) were matched in pairs with healthy cows (NALRT), based on their lactation day (difference in DIM between pairs in early lactation ± 1 day, in high lactating cows ± 5 days) and the number of lactations. Healthy NALRT cows had to meet the criteria of rectal temperature ≤ 39.5 °C, a body condition score within a range of 2.5–4.0 according to Edmonson et al. [26], and a lameness score ≤ 2 according to Sprecher et al. [27]. A health alert was considered valid if it persisted for at least 12 h. The rumen fluid sample was collected within the first 12 h of the health alert.

### 2.4. Rumen Fluid Collection

Rumen fluid was taken twice by two veterinarians using an oral stomach tube (SELEKT Rumen Fluid Collector, Nimrod Veterinary Products, Gloucestershire, United Kingdom). The first collection of rumen fluid (TIME1) was performed within 12 h after the onset of the alert, and the second collection of rumen fluid (TIME2) within 24 h after the end of the alert. To minimize the influence of diurnal variation, the rumen fluid of TIME2 was collected at the same time of the day as that of TIME1. For this reason, the time interval of 24 h after the health alert was set. The rumen fluid samples were collected six hours after the feed supply in the morning, if the additional time criteria were met. The sampling steps were performed according to the guidelines of the SmartCow consortium [28]. Considering consistent insertion depths of the oral stomach tube, a marker was set at the length of 1.8 m. The order of rumen fluid collection was randomized for matched pairs of cows (ALRT vs. NALRT) by starting with the animal with a lower animal identification number. After discarding the first 500 milliliter of each rumen fluid sample, the following 400 milliliter were collected into a measuring cup (2 L MS Water Solutions, Schippers GmbH, Kerken, Germany). To minimize the effect of rumen fluid temperature on the results, the measuring cup was placed in a bucket of warm water (approx. 36 °C) during rumen fluid collection. The collected rumen fluid samples were stored in a thermal cup (GRÄWE thermal cup 0.4 L, Günter Gräwe GmbH, Lüdenscheid, Germany) and immediately transferred to the examination room, which was located on the dairy farm.

### 2.5. Rumen Fluid Examination

In a preliminary experiment, rumen fluid was collected from 18 cows (9 ALRT cows and 9 NALRT counterparts) for two months to train the principal observer (A.S.) in the rumen fluid collection procedures and examination steps.

**Figure 1:**
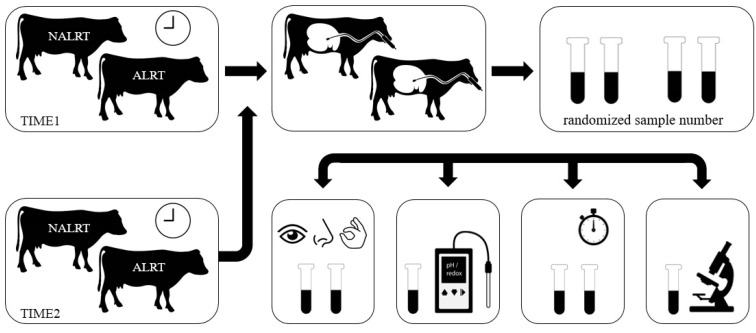
Study design. Cows with a health alert (ALRT) were matched with healthy cows (NALRT) to sample rumen fluid twice daily at the same time, first within 12 h after the start of the alert (TIME1) and second within 24 h after the end of the alert (TIME2). The animal’s alert status was blinded through a randomized sample number before the examination of rumen fluid parameters. For the rumen fluid examination, the content of 20 milliliter of rumen fluid was filled into two test tubes, for each animal. Rumen fluid parameters were assessed for color, odor, consistency (between thumb and forefinger), rumen pH, redox potential, sedimentation flotation time, and the number of protozoa.

Rumen fluid samples were collected to study the parameters of color, odor, consistency, rumen pH, redox potential, sedimentation flotation time, and protozoa in number. The cow’s alert status (ALRT vs. NALRT) at sampling was blinded before the examination by replacing the animal’s identification number with a randomly assigned sample number by a second person. Consequently, the principal observer, who examined all rumen fluid samples did not know the alert status of the cows from which the samples were obtained. Aliquots of 20 mL of each sample were placed into two test tubes (test tube 35 mL, Paul Marienfeld GmbH & Co. KG, Lauda-Königshofen, Germany). Afterward, the samples were placed in a water bath (W350 E, Memmert GmbH + Co. KG, Schwabach, Germany) at 38–39 °C to standardize the temperature of rumen fluid samples for further examinations. Before each consecutive assessment of the parameters, the test tube was gently inverted. First, a sensory assessment of color, odor, and consistency was performed [4]. A scheme of a European color management system (RAL; 705030, 705055, 755030, 805030, 905040, 955040) was used to determine the color of the rumen fluid. The odor of the rumen fluid was classified into three qualities “sour”, “foul”, and “aromatic to fade”, where “aromatic to fade” corresponded to the physiological expectation. For the organoleptic evaluation of the consistency of “watery”, “watery to viscous”, and “viscous to foamy”, an aliquot of rumen fluid was placed between the examiner’s thumb and forefinger. The pH and redox potential were measured using a portable electronic pH meter equipped with a temperature sensor, pH electrode, and redox electrode (G 1501 Series, GHM GROUP Greisinger, Regenstauf, Germany; pH electrode GE 114-WD; redox electrode GR 175 BNC). Both electrodes were marked to ensure a uniform depth in the rumen fluid samples. The sedimentation flotation time test was performed according to the methods described by Dirksen and Smith [29], using one of the two test tubes as a reference to determine the start and end of the test, and using a timer (Digital Timer, TFA Dostmann GmbH & Co KG, Wertheim-Reicholzheim, Germany) for the duration of the test. For the microscopic evaluation of protozoa, 20 microliters of rumen fluid was added to a counting chamber (Fuchs-Rosenthal, Paul Marienfeld GmbH & Co.KG, Lauda-Königshofen, Germany). The average number of protozoa in two of four main quarters was determined at 40× magnification (Olympus CX21, Olympus Europa SE & Co. KG, Hamburg, Germany). Figure 1 shows a schematic presentation of the study design. All findings were documented on a worksheet and transferred to an Excel spreadsheet (MS Excel, Microsoft Excel, version 2102) by the principal observer.

### 2.6. Statistics

Data were imported into the SPSS statistical software package (SPSS version 27.0.0.0, IBM Corporation SPSS Statistics) for analysis. All rumen fluid parameters were tested for normal distribution by the Kolmogorov–Smirnov test.

Based on the pH values of cows with a health alert (mean ± standard deviation: 6.80 ± 0.178) and their healthy counterparts (mean ± standard deviation: 6.58 ± 0.275) in the preliminary experiment with an effect size of 0.948, a two-tailed *t*-test was performed to determine sample size (α = 0.05, β = 0.2, power = 0.8; G*power Version 3.1.9.6, Heinrich Heine University Düsseldorf). Based on this, 20 pairs of cows per lactation stage were required to detect a significant difference in rumen fluid parameters between ALRT and NALRT animals.

To determine the intra-observer agreement of repeated rumen fluid samplings Cohen’s kappa (ҡ) was calculated for ordinal data, and the rho correlation coefficient (rs) according to Spearman was calculated for continuous data. For testing the distribution of lactation stages and odor qualities among cows, the chi square test based on Pearson was performed.

The objective of this study was to determine potential variations in rumen fluid parameters between alert (ALRT) and non-alert (NALRT) groups at two separate sampling times (TIME1 and TIME2). To achieve this, a Mann-Whitney U-test was performed. To detect within-group changes in rumen fluid parameters between TIME1 and TIME2, a related-samples Wilcoxon signed-rank test was conducted. A *p*-value of *p* ≤ 0.05 was considered significant.

A mixed model of repeated measurements was complementary performed to analyse the influence of the interaction between group, rumen fluid collection time, and lactation stage on rumen fluid parameters. The fixed factors included in the model were the alert status (ALRT vs. NALRT), the lactation stage (early lactation (eL) vs. mid to late lactation (mlL)), and the interaction between alert status, examination time, and lactation stage. The individual animal was considered a random factor. Data of rumen fluid parameters that did not meet the requirements of normality were converted into normal distribution by the use of logarithmic or inverse transformation to carry out the mixed model.

A precise definition for the assignment of the cows was determined according to the lactation period and the alert status. The following terms combine the status of alert and early or mid to late lactation of the animals and were used in the further analysis: ALRT_eL; NALRT_eL; ALRT_mlL; NALRT_mlL. If not indicated differently, the averages are reported as mean ± standard error (SEM), with a 95% confidence interval.

## 3. Results

During the study period, rumen fluid was collected from 63 pairs of multiparous cows (*n* = 20 eL; *n* = 43 mlL). Twelve pairs were excluded from statistical analysis because the duration of the health alert was less than 12 h (*n* = 1 eL; *n* = 4 mlL), with no possibility of a second rumen fluid collection (*n* = 2 eL; *n* = 3 mlL), and the onset of a health alert in the associated control (i.e., NALRT) animal (*n* = 2 eL). In total, 15 pairs of cows in eL with 3.2 ± 0.2 lactations and 36 pairs of cows in mlL with 3.4 ± 0.2 lactations were used for the statistical analyses. The distribution of the number of lactations did not differ between lactation stages (*p* = 0.98). For each cow, rumen fluid samples were collected and examined twice (TIME1 and TIME2), resulting in 204 samples in total.

### 3.1. Case Selection and Intra-Rater Agreement

Based on 20 random samples, the intra-rater agreement for the principal observer, who examined all rumen fluid samples, was evaluated (Table 2). For the ordinal sensory characteristics, the agreement for color, odor, and consistency ranged between ҡ ≥ 0.70 and 0.84, and Spearman’s rs = 0.60 and 0.86. The remaining metric variables showed the reliability of agreement in a range of rs = 0.89 and 0.98.

### 3.2. Description of Health Alert and Rumen Fluid Collection

The mean duration of health alerts did not differ between both lactation stages (55 ± 14 h eL; 46 ± 8 h mlL; *p* = 0.97). All cows in early lactation experienced TIME1 within the first two days in milk (mean: 0.8 ± 0.1 DIM, minimum: 0 DIM, maximum: 2 DIM), whereas 50% of the cows in mid to late lactation had TIME1 later than 132 DIM (mean: 165.4 ± 12.2 DIM, minimum: 21 DIM, maximum: 403 DIM). The mean interval between the beginning of a health alert and the collection of rumen fluid was shorter (9 ± 6 h eL; 7 ± 3 h mlL) than the mean interval between the end of the alert and rumen fluid collection (14 ± 6 h eL; 13 ± 6 h mlL). This implies that cows had the second rumen fluid collection 66 ± 14 h (eL) and 58 ± 8 h (mlL) after the onset of the health alert. The exact time of day of TIME1 and TIME2 varied by 15 ± 4 min for cows in eL and 13 ± 2 min for cows in mlL.

### 3.3. Rumen Fluid Parameters

**Figure 2:**
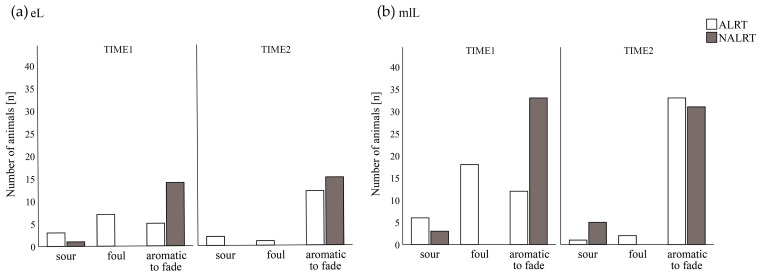
Distribution of odor qualities (sour, foul, aromatic to fade) at the first (TIME1) and second (TIME2) rumen fluid collections according to the health alert (ALRT vs. NALRT): (**a**) 15 pairs of cows in early lactation (eL); (**b**) 36 pairs of cows in mid to latelactation (mlL).

Considering ALRT cows of both lactation stages at TIME1, we found different values (*p* < 0.01) for the parameters of odor (Figure 1), rumen pH, redox potential, sedimentation flotation time, and the number of protozoa (Figure 2) for the ALRT cows compared with their NALRT counterparts. For cows at early lactation, no difference between ALRT_eL and NALRT_eL at TIME1 was found for the rumen fluid parameters of redox potential and the number of protozoa.

No differences were detected between ALRT and NALRT animals at TIME2 of both lactation stages. When analyzed separately, the number of protozoa between ALRT_mlL and NALRT_mlL cows differed (*p* = 0.01) also at TIME2. Detailed information on the descriptive data (mean ± SEM, median, and interquartile range (IQR)) of ALRT and NALRT categorized into subgroups at both rumen fluid collection times is presented in Table 3.

**Figure 3:**
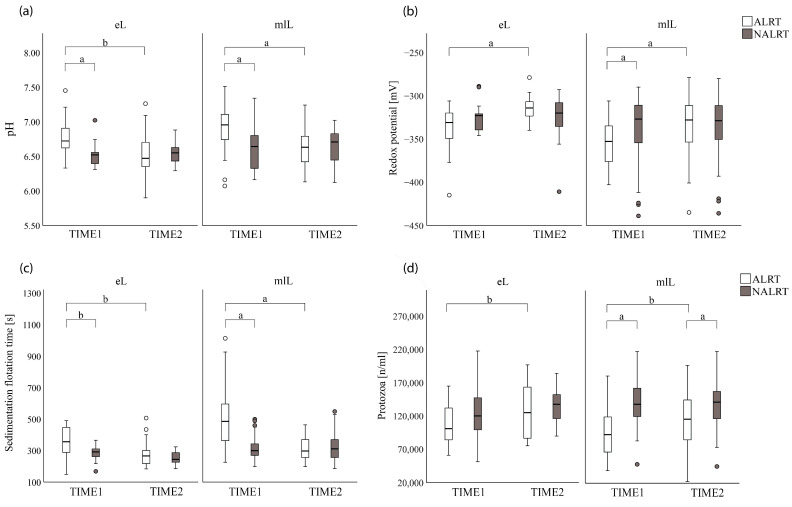
Boxplots of the distribution of rumen fluid parameters for cows with a health alert (ALRT) and their counterparts (NARLT) in early and mid to late lactation (eL and mlL) at the first (TIME1) and second (TIME2) rumen fluid collections: (**a**) rumen pH; (**b**) redox potential (millivolt); (**c**) sedimentation flotation time (seconds); (**d**) the number of protozoa per milliliter of rumen fluid. Significant differences are presented with a: *p* < 0.01, b: *p* < 0.05.

Furthermore, the rumen fluid parameters of all ALRT cows differed between the two rumen fluid collection times (*p* < 0.01) for rumen pH, redox potential, sedimentation flotation time, and the number of protozoa, whereas the rumen fluid parameters for NALRT cows showed no differences between the two collection times (Figure 3).

Consequently, the mixed model for repeated measurements confirmed that all included rumen fluid parameters were affected by the interaction between alert status (ALRT, NALRT) and examination time (*p* < 0.01; *p* = 0.03 for protozoa). Except for the redox potential, the status of health alert, as a single factor, affected the rumen fluid parameters of pH, sedimentation flotation time, and the number of protozoa (*p* < 0.01, for all). In addition, the rumen pH (*p* = 0.05), redox potential (*p* = 0.04), and sedimentation flotation time (*p* < 0.01) were affected by the lactation stage. None of the rumen fluid parameters was influenced by the interaction between alert status and lactation stage. Table 4 presents the differences in rumen fluid parameters between cows with a health alert and their healthy counterparts at both times of rumen fluid collection as well as the influence of the fixed factors of the mixed models. The variability of rumen fluid parameters stratified by the individual animal as a random factor ranged from 6% for redox potential and sedimentation flotation time, 14% for rumen pH, to 31% for the number of protozoa.

## 4. Discussion

Rumen fluid analysis as well as assessment of rumination activity indicate changes in rumen function and associated disorders. Hence, it is considered a predictor for other diseases and metabolic disorders. This study aimed to investigate the association between selected rumen fluid parameters and sensor-based health alerts in dairy cows in early and mid to late lactation.

The physiological range of rumen pH taken by an oral stomach tube has been described between pH 5.5 and 7.2 [8,29]. Deviations indicate disorders such as rumen alkalosis, acidosis, and rumen dysfunction [30,31]. In our study, the mean pH values for ALRT and NALRT cows at both lactation stages at the two rumen fluid collection times were within the physiological pH range. Surprisingly, ALRT cows had greater pH values during health alerts than their assigned control animals. On the contrary, the meta-analysis of Souza et al. [32] on the variation in rumination behavior related to milk, and variables mechanistically associated with milk fat synthesis, described a linear relationship between the increase of rumination time per day and the increase of rumen pH. Souza et al. [32] also limited their analysis of variables to a sum per day but did not consider the diurnal variation of rumen pH and rumination time. Sensor-based systems can detect variations in the diurnal rumination time of individual animals within hours. This more narrowly defined approach could lead to a different association of rumination time and rumen pH. Furthermore, ALRT cows in our study experienced greater pH variations between TIME1 and TIME2 than their NALRT partner animals. Since diurnal patterns of consciously monitored reticulorumen pH are predictable [33] and the exact time of day of rumen fluid collections in this study varied only between 13 ± 2 to 15 ± 4 min, the greater pH variations experienced by ALRT animals seem to be associated with health alerts. Greater pH variations affect rumen function [34,35] and therefore, demonstrate a vulnerable status of rumen pH. Geishauser et al. [36] stated that the cow’s age, day of lactation, as well as diurnal fluctuations [37], are factors that influence rumen pH. Our study attempted to minimize these influencing factors by using an appropriate study design. We matched pairs of cows with the same number of lactation and DIMs as best as possible and collected both rumen fluid samples at the same time of the day. Given the study design and the smaller variance of rumen pH for NALRT cows during the study period, we assume the results of rumen pH to be associated with the health alerts.

The redox potential of the rumen fluid reflects the status of the microbial intracellular redox balance and impacts the fermentative activity of rumen microorganisms [7,37]. In our study, the Mann–Whitney U-test only showed a difference in redox potential between ALRT_mlL and NALRT_mlL cows at TIME1. The status of health alert as a single factor did not affect the redox potential in the mixed models, but the interaction between the status of health alert and examination time did. The different conclusions between the Mann–Whitney U-test and the mixed models of repeated measurements could be attributable to the different group sizes of cows in early lactation (*n* = 15) and cows in mid to late lacatation (*n* = 36). Marden et al. [38] described oxygen as a leading factor for a shift in the redox equilibrium. The sampling method of rumen fluid in our study included contact with oxygen before the measurement of the redox potential by an electrode at both examination times. Although all rumen fluid samples were exposed to oxygen before the measurement of redox potential, the interaction between health alert and examination time as a factor affecting the redox potential could be influenced by a shift in the redox equilibrium. In conclusion, no clear association between health alerts and redox potential was detectable.

The microbial activity of the rumen fluid was determined by the number of protozoa and the sedimentation flotation time, which is related to the protozoal activity [29]. The physiological range of protozoa in rumen fluid is reported from10³ to 10⁶ per mL [39]. In this study, the numbers of protozoa were within the reported range, but smaller numbers were found for ALRT_mlL animals at TIME1 compared with their NALRT_mlL counterparts. In agreement with these findings, the sedimentation flotation time was prolonged for ALRT_mlL cows at TIME1 compared to NALRT_mlL. The instability of the rumen microflora caused by smaller numbers of protozoa is related to greater diurnal pH fluctuations [34,40]. The difference in the number of protozoa between ALRT_mlL and NALRT_mlL cows persisted after the end of the health alerts. Assuming that protozoan regeneration is a continuous process, the protozoa in our study were unable to reproduce fully until TIME2, which would justify the remaining difference between ALRT and NALRT animals. For ALRT_eL cows there was only a difference between rumen fluid collection times detectable. All ALRT cows in early lactation experienced the health alert within two days after calving. The lower numbers of protozoa for those cows independent of their status of rumination and the missing differences in the number of protozoa at TIME1 might be caused by the overlapping effect of a general adaptation of protozoa to the altered diet composition after calving [41].

The stage of lactation, eL and mlL, affected the parameters of rumen pH, redox potential, and sedimentation flotation time in the mixed models for repeated measurements. Considering that cows in early lactation face metabolic changes associated with calving, changes in diet, and the sudden increase in milk production [16,17,42], different ranges of the measured parameters for rumen fluid parameters were to be expected. Although the lactation stage had an impact on the measured values of the mentioned rumen fluid parameters, no effect on the interaction between the lactation stage and alert status (ALRT vs NALRT) was observed. Consequently, cows in early and mid to late lactation did not differ in rumen fluid characteristics.

Rumen fluid sampling via an oral stomach tube represents only a snapshot of rumen physiology in cows with different rumination times and does not necessarily reflect dynamic processes. Disadvantages of sampling with an oral stomach tube also include the variable and unpredictable position of the tube in the rumen and possible contamination with saliva [31,43,44]. To minimize these disadvantages, an oral stomach tube previously evaluated by Steiner et al. [44] was chosen in this study. The authors did not report differences in the pH between rumen fluid samples collected by the tube or collected from fistulated cows.

This study demonstrated a change in the rumen fluid parameters of odor, rumen pH, sedimentation flotation time, and protozoan count associated with health alerts. In particular, this study demonstrated that health alerts are associated with increased variability in rumen fluid parameters. The association between health alerts and rumen function parameters presents a promising fact for early disease detection. For a comprehensive assessment of rumen physiology processes, further studies should include continuous measurements of rumen fluid parameters at diurnal rumination times and evaluate the clinical significance of those sensor-based health alerts.

## 5. Conclusions

The rumen fluid parameters of odor, rumen pH, sedimentation flotation time, and protozoan count of cows with sensor-based health alerts differed from the parameters of their healthy counterparts during the health alert. Although these differences were within physiological ranges, cows with health alerts showed significant variation in those selected parameters between the two sampling times. The rumen fluid parameters of their NALRT counterparts remained constant. Furthermore, all rumen fluid parameters were affected by the interaction between health alert and examination time in the mixed models. No effect was found for the interaction between health alert and lactation stage, even though the lactation stage as a single factor affected the measured parameters of the rumen fluid parameters. In summary, an association between sensor-based health alerts and rumen physiology was found, especially greater pH values, fewer numbers of protozoa, and an extended duration of sedimentation flotation time for cows with health alerts.

## Figures and Tables

**Table 1:** Composition of dietary ingredients and nutrients of the total mixed ration (TMR) for cows in early and mid to late lactation.

Feed Ingredients or Nutrients	TMR Fed in the Study Period	
	Early Lactation	Mid to Late Lactation
DMI ¹ per cow (kg)	21	25
Dry matter (DM, %)	40.9	40.7
Ingredients (% of DM)		
Agf 2 me ²	6.5	7.6
Corn mix	4.8	5.6
Corn-Cob-Mix	5.4	6.4
Corn silage	23.7	25.4
Grass silage	26.5	24.6
Forage rye silage	10.0	9.3
Rape seed meal	3.6	4.3
Rape expeller	5.6	6.6
Soybean hulls	1.2	1.4
Soybean meal	4.6	5.3
Vinasse	2.8	3.3
Chopped straw	2.2	
Fresh cow 1 ^3^	3.0	
Nutrients (g per kg DM)		
Net energy lactation (MJ)	6.92	7.09
Crude protein	158	167
Crude fat	38	34
Crude fiber	190	180
Starch	125	140
Sugar	36	38
Calcium	10.07	8.43
Phosphorus	4.08	3.99
Potassium	18.47	18.54
Magnesium	3.4	3.13
Sodium	5.94	4.87

g: gram; MJ: megajoule; ¹ DMI (calculated dry matter intake per cow by feed management system (TMR Tracker™ version 5.3.0.531, Topcon, WI, USA)). ² Agf 2 me (dietary supplement for dairy cows that contains vitamins and minerals). ^3^ Fresh cow (dietary supplement for cows in early lactation which contains vitamins and minerals).

**Table 2:** Intra-observer reliability for evaluated rumen fluid parameters: Cohen’s kappa (ҡ) and Spearman correlation (rs).

Rumen Fluid Parameters	Number of Samples	ҡ	rs
Ordinal data			
Color	20	0.84	0.86
Odor	20	0.77	0.60
Consistency	20	0.70	0.83
Metric data			
Rumen pH	20		0.98
Redox potential	20		0.96
Sedimentation flotation time	20		0.97
Protozoa in number	20		0.98

**Table 3:** Rumen fluid parameters of cows with a health alert (ALRT) and their healthy counterparts (NALRT) at both lactation stages (eL and mlL) at the first rumen fluid collection (TIME1) within 12 h of the start of the health alert and the second rumen fluid collection (TIME2) within 24 h after the end of health alert.

Repeated Measurements	*N*	TIME1			TIME2		
		Mean ± SEM	Median	IQR	Mean ± SEM	Median	IQR
Rumen pH							
ALRT_eL	15	6.78 ± 0.08	6.72	0.39	6.55 ± 0.09	6.47	0.44
NALRT_eL	15	6.53 ± 0.05	6.52	0.17	6.54 ± 0.04	6.55	0.21
ALRT_mlL	36	6.92 ± 0.05	6.96	0.40	6.63 ± 0.04	6.63	0.38
NALRT_mlL	36	6.58 ± 0.05	6.64	0.49	6.64 ± 0.04	6.73	0.38
Redox potential (mV)							
ALRT_eL	15	−339 ± 8	−331	33	−319 ± 6	−314	19
NALRT_eL	15	−329 ± 7	−323	19	−327 ± 8	−320	39
ALRT_mlL	36	−357 ± 5	−353	44	−338 ± 7	−328	45
NALRT_mlL	36	−339 ± 7	−327	44	−334 ± 6	−329	40
Sed.Flot. time * (s)							
ALRT_eL	15	429 ± 70	355	208	282 ± 24	265	100
NALRT_eL	15	284 ± 13	291	59	254 ± 11	244	61
ALRT_mlL	36	511 ± 36	485	241	316 ± 13	297	115
NALRT_mlL	36	321 ± 15	299	77	327 ± 20	310	115
Protozoa (*n* × 10^3^/mL)							
ALRT_eL	15	108 ± 8	101	51	127 ± 11	125	94
NALRT_eL	15	123 ± 10	120	51	135 ± 6	138	42
ALRT_mlL	36	96 ± 6	93	55	114 ± 7	116	62
NALRT_mlL	36	141 ± 6	138	44	140 ± 6	141	42

*n*: number of animals; mV: millivolt; s: seconds; *n* × 10^3^/mL: number of protozoa per milliliter; * Sedimentation flotation time.

**Table 4:** Mean ± SEM of the repeated measurements of rumen fluid parameters for cows with a health alert (ALRT) and their counterparts (NALRT) at the first and second rumen fluid collections (TIME1 and TIME2) as well as the effects of alert status (ALRT vs. NALRT), stage of lactation (LS), and interactions of alert status × rumen fluid collection time (RF time) and alert status × LS.

Measurements, Groups	*n*	Rumen Fluid Collection		*p*-Value			
		TIME1	TIME2	Alert Status	LS	Alert Status × RF Time	Alert Status × LS
Rumen pH				< 0.01	0.05	< 0.01	0.74
ALRT	51	6.87 ± 0.32 ^a^	6.61 ± 0.29 ^b^				
NALRT	51	6.57 ± 0.26	6.61 ± 0.23				
*p*-value		<0.01	0.84				
Redox potential (mV)				0.30	0.04	< 0.01	0.36
ALRT	51	–352 ± 32 ^a^	–333 ± 39 ^b^				
NALRT	51	–336 ± 37	–332 ± 35				
*p*-value		<0.01	0.79				
Sed.Flot. time * (s)				< 0.01	< 0.01	< 0.01	0.85
ALRT	51	487 ± 234 ^a^	306 ± 83 ^b^				
NALRT	51	310 ± 80	306 ± 107				
*p*-value		<0.01	0.75				
Protozoa (*n* × 10^3^/mL)				< 0.01	0.96	0.03	0.08
ALRT	51	99 ± 36 ^a^	118 ± 42 ^b^				
NALRT	51	136 ± 37	138 ± 34				
*p*-value		<0.01	0.01				

*n*: number of animals; mV: millivolt; s: seconds; *n* × 10^3^/mL: number of protozoa per milliliter. * Sedimentation flotation time; ^a,b^ means within rows differ (*p* < 0.01).

## Data Availability

Not applicable.
